# Rare case of concurrent suprasternal and cardiac metastasis from small bowel neuroendocrine tumour

**DOI:** 10.1093/jscr/rjac308

**Published:** 2022-06-22

**Authors:** Xinyi Nan, Anoj Dharmawardhane

**Affiliations:** Surgical & Critical Care Division, Gold Coast Health & Hospital Services, Gold Coast, Queensland, Australia; Department of Surgery, Darling Downs Hospital & Health Services, Toowoomba, Queensland, Australia

## Abstract

Neuroendocrine tumours (NETs) are rare tumours derived from the neuroendocrine cell system, arising across a wide range of organs, most commonly the gastrointestinal tract and bronchopulmonary symptoms. Although NETs can metastasis widely throughout the body, cardiac metastasis is rare with an incidence of 2–4% and usually presents in the presence of extensive metastasis elsewhere. Suprasternal metastasis to the neck is exceedingly rare with <20 cases reported in the literature. We report the case of a 71-year-old female with concurrent cardiac and suprasternal metastasis at diagnosis of terminal ileal NET.

## INTRODUCTION

Neuroendocrine tumours (NETs) are rare tumours derived from the neuroendocrine cell system, arising across a wide range of organs such as gastrointestinal (GI) tract, bronchopulmonary system and thymus [1]. Constituting only 0.5% of all malignant conditions and 2% of all malignant tumours of the GI tract, majority of them are found in the small intestine. Given the mostly slow growing nature of NET and its wide variability in clinical presentation, about one-fifth of patients present with metastatic disease at diagnosis [2, 3]. Although NETs can metastasise widely throughout the body, cardiac metastasis (CM) is rare, and even less so are reported cases of neck metastasis. We report a case of concurrent cardiac and neck metastasis from terminal ileal NET.

## CASE REPORT

A 71-year-old female underwent colonoscopy for a 12-month history of diarrhoea, which revealed abnormal ileocecal mucosa. Computed tomography (CT) chest abdomen and pelvis was suggestive of primary appendiceal malignancy with soft tissue expansion and a midline soft tissue mass just inferior to the thyroid. She underwent laparoscopic right hemicolectomy that revealed a Stage pT2N2 16-mm Grade 1 NET in the terminal ileum with 5/24 lymph node involvement, mitotic rate < 2/mm^2^ and Ki67 < 1%.

**Figure 1:**
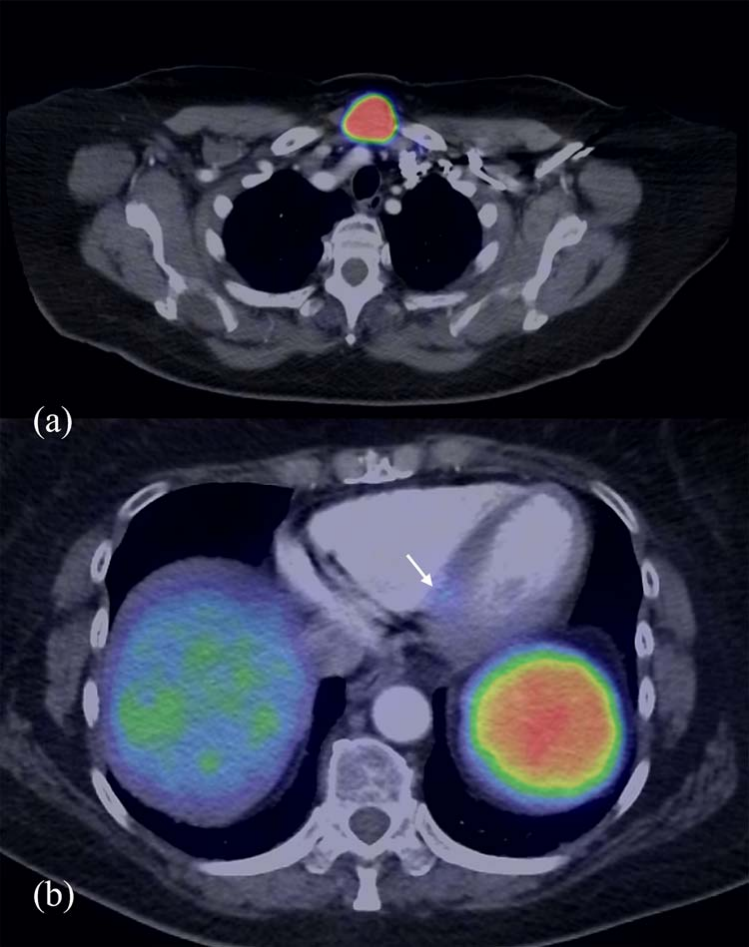
DOTA PET/CT demonstrating avidity in the midline neck mass (**a**) and the left ventricular septum inferiorly (**b**).

Post-operative staging DOTA positron emission tomography (PET)/CT (Fig. 1) revealed again a 33 × 26 mm midline neck mass just above the manubrium, which was intensely FDG avid, and a mild avid 5-mm node posterior to the left medial clavicle. A focus of activity was also noted in the left ventricular septum inferiorly along with suspicious avid left inguinal node. The neck mass was initially thought to be of thyroid origin as a fine needle aspiration showed a Category 4 follicular neoplasm. Formal surgical excisions found instead a 2.5-cm superficial cystic mass in central anterior neck, deep to the investing layer of deep cervical fascia and adherent to bilateral clavicles with no thyroid involvement. Histology confirmed metastatic NET, Grade 1 Ki67 < 2% with similar morphology to the ileal primary.

Cardiac magnetic resonance imaging (MRI) revealed a 3-mm focus of increased STIR signal at the proximal inferoseptum corresponding to the location of previous focal DOTATATE uptake, confirming a metastatic interventricular septum lesion. There was otherwise normal left ventricular systolic function with an EF of 70%. The patient remained largely asymptomatic aside from occasional flushing sensation and loose motions for three to four times a day, which was stable since the right hemicolectomy. She did not have any heart failure symptoms. She was commenced on lanreotide treatment at 120-mg monthly, despite being relatively asymptomatic, due to the presence of CM.

Surveillance included regular chromogranin A, which decreased rapidly from 418 to 269 post-resection of primary ileal disease and suprasternal metastasis and returned to within normal range (20–102) 7 months post-resection. Yearly cardiac MRI showed stable cardiac disease. Three new hepatic metastases were found on surveillance PET, 18 months post-resection, however, the patient remained well with no carcinoid symptoms. She continues lanreotide and regular 6 monthly PET and yearly cardiac MRI surveillance.

## DISCUSSION

CM from NETs is rare, with incidence estimated to be 2–4% [4]. Variance is noted within literature, ranging from 0.8% in a large case series, up to 13% in another retrospective analysis [5, 6]. The latter’s higher prevalence may be a result of achieving early detection of small asymptomatic lesions with the use of more sensitive imaging modalities like 68Ga-DOTATOC and 18F-FDOPA.

A wide range of clinical presentations is seen with CM, from asymptomatic to fulminant heart failure, obstructive/cardiogenic shock and cardiac arrests [7]. Location and size are key determinants of symptoms. A review of literature for anatomical distribution of CM (*n* = 124, Table 1) showed left ventricle (36.3%) to be the most common location, followed by right ventricle (29%) and septum (16.9%). CMs are generally detected in patients with high tumour burdens, carcinoid syndrome and widespread metastatic disease, with few reported to present without liver involvement [6–9]; in our patient, the CM remained stable with liver metastasis only developing at over 18 months of post-initial diagnosis and primary resection.

**Table 1:** Distribution of cardiac metastases (total *n* = 124) locations

	LV	RV	Septal	Bilateral	LA	RA	Pericardial
Ghannudi *et al*. [12]	1	-	1	-	-	-	-
Jann *et al*. [13]	11	10	7	10	-	-	-
Kunz *et al.* [14]	9	3	9	-	-	-	-
Liu *et al.*	13	11	3	-	1	1	4
Noordzij *et al.*	3	6	-	6	-	-	-
Pandya *et al.*	8	6	1	-	-	-	-
**% as total**	**36.3%**	**29%**	**16.9%**	**12.9%**	**0.8%**	**0.8%**	**3.2%**

LV = left ventricle, RV = right ventricle, LA = left atrium, RA = right atrium.

It is important to distinguish between carcinoid heart disease (CHD) and CM from NET. CHD can occur in up to 50–60% of patients with carcinoid syndrome and is characterized by the endocardial deposition of plaque-like fibrous tissue commonly on valvular cusps, papillary muscles and cords, with a predilection involving the right side of the heart [10]. It manifests as valvular dysfunction and progressive heart failure. CM appears as a separate entity, with an analysis showing its presence to be not related to the occurrence of CHD, although they certainly may coexist at the same time [6, 7, 9].

The presence of suprasternal metastasis from confirmed GI NET is exceedingly rare; true incidence is not clear as historically cervical and mediastinal metastases have been regarded as medically insignificant. Less than 20 cases have been reported from 1975 until now, the majority of these in a study by Wang YZ *et al*. who demonstrated an incidence of up to 8.7% [11]. This number, higher than previously accepted in the field (3–4%), has not been confirmed by large population studies yet. Anatomical location of neck metastases can give rise to various compressive symptoms due to surrounding neurovascular structures and the aerodigestive tract. Biochemically, it can also act as a source of serotonin secretion contributing to carcinoid syndrome.

To the best of our knowledge, this is the first reported case of concurrent cardiac and neck metastasis from small bowel NET. Importance of early DOTA PET imaging is highlighted for the detection of uncommon metastatic sites. There is no clear consensus on the management of CM and their response to treatment modalities; regular DOTA PET/CT in combination with cardiac MRI for monitoring appears to be the gold standard. However small and asymptomatic CM may be, its presence can sway management to commence somatostatin analogue despite minimal symptoms and low volume disease post-resection. Early surgical excisional biopsy of neck metastasis, rather than relying on FNA, is key in obtaining an accurate tissue diagnosis, removing the potential source of serotonin secretion and eliminating the potential for developing compressive symptoms later.

## CONFLICT OF INTEREST STATEMENT

None declared.

## FUNDING

None.
